# Experience of traumatic events in people with severe mental illness in a low-income country: a qualitative study

**DOI:** 10.1186/s13033-023-00616-4

**Published:** 2023-12-05

**Authors:** Lauren C. Ng, Kimberly Hook, Maji Hailemariam, Medhin Selamu, Abebaw Fekadu, Charlotte Hanlon

**Affiliations:** 1 Present Address: Department of Psychology, University of California Los Angeles, Los Angeles, CA USA; 2 Department of Psychiatry, Boston Medical Center, Boston, MA USA; 3 Department of Psychiatry, Boston University, School of Medicine, Boston, MA USA; 4 Department of Psychiatry, Massachusetts General Hospital, Boston, MA USA; 5 Present Address: Department of Epidemiology, Harvard T.H. Chan School of Public Health, Boston, MA USA; 6 Department of Obstetrics, Gynecology and Reproductive Biology, College of Human Medicine, Michigan State University, East Lansing, MI USA; 7 Present Address: Charles Stewart Mott Department of Public Health, College of Human Medicine, Michigan State University, Flint, MI USA; 8 Present Address: Center for Innovative Drug Development, Therapeutic Trials for Africa (CDT-Africa), Addis Ababa University, School of Medicine, College of Health Sciences, Addis Ababa, Ethiopia; 9 Present Address: Department of Psychiatry, School of Medicine, College of Health Sciences, Addis Ababa University, Addis Ababa, Ethiopia; 10 Health Service and Population Research Department, Centre for Global Mental Health, King’s College London, WHO Collaborating Centre for Mental Health Research and Training, Institute of Psychiatry, Psychology and Neuroscience, London, UK

**Keywords:** Severe mental illness (SMI), Trauma; Low- and middle-income countries (LMICs), Quality of life

## Abstract

**Background:**

This study describes the trauma experiences of people with severe mental illness (SMI) in Ethiopia and presents a model of how SMI and trauma exposure interact to reduce functioning and quality of life in this setting.

**Methods:**

A total of 53 participants living and working in a rural district in southern Ethiopia were interviewed: 18 people living with SMI, 21 caregivers, and 14 primary health care providers.

**Results:**

Many participants reported that exposure to traumatic and stressful events led to SMI, exacerbated SMI symptoms, and increased caregiver stress and distress. In addition, SMI symptoms and caregiver desperation, stress or stigma were also reported to increase the possibility of trauma exposure.

**Conclusions:**

Results suggest it is incumbent upon health professionals and the broader health community to view trauma exposure (broadly defined) as a public health problem that affects all, particularly individuals with SMI.

## Background

Psychological trauma is described as exposure to actual or threatened death, serious injury, or sexual violence, with common examples including exposure to war, physical assault, sexual trafficking, and domestic violence, which can lead to psychological harm [1]. Globally, it is estimated that over 70% of people have experienced a traumatic event [2]. However, researchers have suggested that other experiences, such as emotional neglect or humiliation, may also result in psychological harm [3], potentially resulting in even higher rates of affected individuals. Individuals diagnosed with mental illness, and particularly those with severe mental illness (SMI; including disorders such as schizophrenia and bipolar disorder) are at elevated odds of experiencing traumatic events when compared to the general population [4–6]. Strikingly, one study conducted in the US found that 98% of participants with SMI experienced at least one traumatic event during their lifetime [7], and a second study similarly finding that 94.3% of women with schizophrenia experienced at least one traumatic event in adulthood [8]. In other studies, individuals with SMI experienced an average of 3.5 traumatic events over their lifetime [7]. Comorbid conditions including substance use disorder may further inflate these rates [9].

Throughout the existing literature, interactions between trauma and mental illness abound, with three relationships of particular note: (a) mental illness increases an individual’s vulnerability to experiencing traumatic events; (b) traumatic events exacerbate mental illness; and (c) traumatic events cause mental illness. Psychotic illness may increase an individual’s likelihood of experiencing a traumatic event due to behaviors stemming from psychotic symptoms [10]. Additionally, for individuals with SMI, experience of psychotic symptoms and its treatment (including hospitalization and use of seclusion and/or restraints) might also be traumatic in and of themselves [11–13]. Traumatic events associated with psychiatric hospitalization include physical and sexual assault, witnessing traumatic events, and being near violent patients [14]. Rates of violence perpetuated against individuals with SMI are particularly high, suggesting again the increased risk of trauma for this group [15, 16]. Trauma experiences, including exposure to multiple trauma events, exacerbate psychiatric symptoms and result in worsened treatment outcomes [10, 17–19]. Such exposure also leads to worsened quality life (such as unstable housing and difficulty accessing services to protect civil rights), increases sensitivity to stress, and results in increased use of acute care services [17, 19–21].

Framed in terms of the diathesis-stress etiological model for psychosis, trauma experiences can be understood as stressors that impact disease onset, progression, and relapse [6, 22, 23]. Cougnard, Marcelis et al. [24] noted that environmental risk factors increase risk of psychosis additively; similarly, early experiences of adversity combine synergistically with recent experiences of trauma to increase the risk of psychosis, while at the same time increasing one’s risk of exposure to later adversity [25].

Traumatic events, particularly childhood sexual abuse, are common in SMI populations and are implicated in later development of mental illness, specifically post-traumatic stress disorder (PTSD) and psychosis [4, 10, 17, 23, 26–28]. Childhood sexual abuse has further been linked to more severe hallucinations, delusions, and intrusive thoughts [29]. Traumatic experiences that occur during adulthood, including experiences such as parental loss of a child, similarly increase risk of subsequent development of psychosis [30]. Other forms of traumatic insult, including bullying, physical abuse, injury, and assault, have also been associated with onset of psychotic symptoms and episodes [31–33]. Experiencing multiple forms of trauma and repeated exposure to traumatic events are also significant predictors of psychosis onset [33, 34].

Almost all of the published research on the relationship between trauma exposure and SMI is derived from high-income countries (HICs), despite the fact that individuals living in low and middle-income countries (LMICs) may face increased rates of trauma exposure due to stressors such as intimate partner violence, sexual assault and exploitation, child labor and marriage, migration and trafficking, and displacement and armed conflict [28, 35] combined with much lower access to mental health services [36]. Further exploration of the intersection of these factors is relevant in Ethiopia, where almost half of rural Ethiopians reported having experienced major threatening events in the previous six months [37]. People with SMI experience high rates of stigma, neglect, chaining and restraint, human rights abuses, physical and sexual violence, and road traffic accidents [38–43], and 9% of people with SMI in Ethiopia die from non-suicide unnatural causes [44]. To address this evidence gap, in this study we aimed to describe the trauma experiences of people with SMI in a rural district in Ethiopia and to present a model of how SMI and trauma exposure interact to reduce functioning and quality of life, particularly in under-resourced settings.

## Methods

This study is a secondary data analysis of interviews conducted in 2015 as part of the Programme for Improving Mental healthcarE (PRIME) project [37, 40, 45]. PRIME was a large-scale, multi-country research program investigating the implementation of integrated district mental health care plans based on task-shared models of care for people with priority mental disorders, including people with SMI. The PRIME study was conducted in Sodo district in the Gurage zone of the Southern Nations, Nationalities and People’s Region of Ethiopia, approximately 100 km from the capital, Addis Ababa. Sodo is 90% rural and most of the 162,000 people live in villages that are geographically spread apart and difficult to access. At the time of this study, Sodo had eight primary care clinics (health centers) staffed by nurses, health officers, and midwives. The number of staff per health center ranged from eight to 24. Approximately 20,000–40,000 people were served by each health center.

As part of PRIME, all providers working in government-owned primary health care clinics were trained to care for people with SMI, depression, epilepsy, and alcohol use disorders using the WHO’s mental health Gap Action Programme (mhGAP) intervention guide, with supervision provided by psychiatric nurses [40, 46]. Community based health extension workers were trained for two days on mental health literacy, case detection, referral, early identification of medication side effects, adherence support, and community awareness raising and outreach to reduce stigma and promote social inclusion. In addition, PRIME provided general mental illness awareness raising to the community [40]. This study is a secondary analysis of interviews conducted to understand barriers and facilitators to accessing task-shared mental health care in the district [44].

### Setting

#### Participants

Individuals with SMI were identified by health extension workers, community leaders, and PRIME outreach workers. Those suspected to have SMI were referred to the nearest primary health care clinic for diagnostic assessment by primary care providers [40]. Individuals who received a diagnosis of “psychosis” or “bipolar disorder” by a primary care worker received a confirmatory clinical interview by a psychiatric nurse using the Operational Criteria for Research (OPCRIT) interview guide [47]. Participants who met the following criteria were recruited into a PRIME intervention cohort study: (a) 18 years or older, (b) planning to stay resident in the district for the next 12 months, (c) provided informed consent or, if they lacked capacity to consent, did not refuse and guardian consent was obtained, (d) had a psychiatric nurse confirmed diagnosis using the OPCRIT, and (e) able to understand Amharic, the official language of Ethiopia and the working language of the study site [48]. Caregivers were eligible to participate if they had lived with someone with SMI for at least four months, were at least 18 years old, were the household head or the older person of two people who contributed equally to household decision making, and if they provided informed consent [49]. For the nested qualitative study, people with SMI and caregivers were purposively selected based on gender, rural/urban residence and the extent of engagement with the task-shared mental health service. Health care workers were recruited from all of the district primary health care clinics, purposively selected based on gender, qualification, and years of experience.

#### Interviews

Written informed consent was obtained from participants. Interviews with service users and caregivers were conducted near their homes at a location of their choice. Interviews with health care providers were conducted at the facility. The interviewers were two female Ethiopian researchers with Masters degrees in Social Work who had previous experience conducting qualitative interviews in Sodo District. All interviews were audiorecorded. The interview topic guide covered experiences of accessing or delivering task-shared care, but also asked about experience of restraint or human rights abuses and economic and social functioning. Since this study was a secondary data analysis, there were no specific questions targeted for coding; instead, the entirety of each interview was reviewed for quotes relevant to the research questions.

#### Data analysis

Interview audiorecordings were transcribed verbatim into Amharic and translated into English. Data were analyzed using NVivo version 11 [50], using an interpretative phenomenological approach. The primary research questions were (a) “What types of traumatic and stressful events do people with SMI experience?” (b) “What are the perspectives of informants on the factors that put people at risk of experiencing traumatic events?” and (c) “What are the consequences of experiencing traumatic events?” Identified traumatic and stressful events were further grouped into those events that met the Diagnosis and Statistical Manual-Version 5’s (DSM-5) criteria for a traumatic event: an event in which someone is directly or indirectly exposed to actual or threatened death, serious injury, or sexual violence [1]. Using open coding, Author 1 generated codes and organized them into a theoretical framework describing potential relationships between severe mental illness and trauma exposure. During data analysis, Author 6 reviewed the data, codes, and the framework. Authors 1 and 6 discussed, adapted, and ultimately agreed on the final codebook and framework. Authors 1 and 2 coded the data and reviewed the results with author 6.

## Results

A total of 53 participants were interviewed, including 18 people living with SMI (5 women), 21 caregivers of people living with SMI (15 women), and 14 male primary health care providers. Most of the 21 caregivers were parents of people living with SMI (*n* = 13), and the rest were spouses (*n* = 3), children (*n* = 3), or siblings (*n* = 2).

### Traumatic events

Descriptions of potentially traumatic events (PTEs) experienced by people with SMI were reported by 31 participants, including 18 of the 21 caregivers and 12 of the 18 people living with SMI. Using the DSM-5 Criterion A definition of a traumatic event, seven different types of PTEs were described, including assaults or beatings, robberies, attacks by animals such as hyenas, dangerous falls such as into ditches or sewage drains, car accidents, drownings, and electric shocks (see Table 1). In addition to traumatic events that met DSM-5 criteria for a traumatic event, participants also described other very stressful and frightening experiences that caused suffering or emotional pain to people with SMI. These included (a) being restrained, tied or shackled, (b) being verbally or emotionally abused, (c) being exploited or disenfranchised, (d) being chased away, and (e) having forced psychiatric treatment or dangerous traditional or religious healing (see Table 2).

**Table 1:** Potentially traumatic events that meet the DSM-5 criterion A definition

Code	Description	Examples
Assault, Beatings	Descriptions of or concerns about the person with SMI being beaten, assaulted or attacked by a person or person(s)	R: “I have faced so many dangers before.… My leg has been broken. To your surprise, whether you believe it or not, this leg of mine was broken…. I: did someone break you? R: Yes, a person from this area. We do not [have] tribal or any other kind of relationship with the people in this area…. But they broke my leg. I have bent it like this… look. Right here. I: Yes. Yes. I saw it. I saw it. R: Right here the bone…. I have bent it this way and stood on it. What is this? I: When people broke your leg. R: Yes. I: It is because they want the land and they are not your relatives. R: Yes. They are not my relatives. I: They want you to drink. They want something to happen to you. R: Yes. Why is that? I do not even have a brother here.”—*Person Living with SMI #5, Male* “I: Have you ever been beaten? R: Yes. My brother beat me one when I disturbed him. He did that when I tried to go around begging for money for alcohol and food. That was after I dropped out of the treatment about five years ago.”—*Person living SMI #4, Female*
Robbery	Descriptions of or concerns about the person with SMI being robbed, mugged, or burglarized	“I: Have other people abused him verbally after he became ill? R: Children abuse him a lot. They take whatever he has in his pocket. They play with him. They try to fool him. They give him alcoholic drinks and pick his pocket. I hear that but we disregard such stories to live in peace with people. I can’t go and confront them because I am old and my health is not so great.”—*Caregiver #7, Father of man living with SMI (previously husband of a woman living with SMI, who has since died)* “R: Their father went to look for our son who got lost for a year. He just walked out of the house and got lost. My husband went to look for him but he was away for long looking for him. We heard that he was around Meki town. He went to different places hoping to find his son. Then he lost all hope and decided to return to his home. Nevertheless, he did not come back. In the meantime, he lost his mind to overthinking. He had some money he took with him when he went to look for his son. He was robbed. After that he became seriously out of his mind. Remember, when the father went to look for the one who was lost, I was at home taking care of our son who was also mentally ill and in chain.”—*Caregiver #4, Mother of a man living with SMI*
Animal attack	Descriptions of or concerns about threat of attack of people with SMI by wild animals	“R: His very first treatment was at Amanuel hospital in Addis Ababa [*the psychiatric hospital in Addis Ababa*]. They told us that he has some mental illness and mental retardation. However, when you look at him he does not act like a mentally retarded person. He is too active for a mentally retarded. His problem is that he does not respond though he understands well. His problem is that he does not foresee the risk. He does not care when it is dark outside and when the hyenas are coming. He does not understand that he has to close the door when it is dark or he has no idea as to when to wake up and open the door.”—Caregiver #10, *Mother of a man living with SMI*
Falls	Descriptions of or concerns about the person with SMI falling into ditches, pits, sewage drains, etc	“R: I got tired… Honestly I came back home because I was tired. I came home. He stayed home after a lot of trial. What can I do? He is sitting at home and I am sitting with him. Where can I take him. Did I not sit at home with him? Since I sat at home… if he went some where he would fall in the sewage. This is because I am the mother… if I was not his mother… I: If you were not… R: Yes. If I was not, what? I: If you were not… R: If I was not, he would have died in one of the sewages. I am suffering to sit with him.”—*Caregiver #9, Mother of a man living with SMI* “I: What are his symptoms now? R: He cannot talk properly. He is very irritable and grumpy around others. He does not want to stay around the neighborhood. He wanders around aimlessly. His spine has a problem. He cannot run if something comes. When he goes around, I worry that he might fall into something.” – *Caregiver #11, Father of a man living with SMI*
Electric shock	Descriptions of or concerns about person with SMI being electrocuted	“I: What do other people say your illness was? R: They think I was doing all these intentionally. I go to them and apologize after insulting people. It wasn’t intentional. I experienced car accident and electric shock. I fell into water because I thought I was God and I had a feeling that the water won’t drown me.—*Caregiver #12, Father of a man living with SMI (secondarily a person living with SMI)*
Hit by vehicle or car accident	Descriptions of or concerns about the person with SMI being in a vehicle accident	“R: He complains that he has mental illness. He says something worries him a lot. It has been around seven years since this has happened. The cause is that he had an accident when he was riding a motor bike to one of the rural villages. He broke his leg and the accident was a major one. The illness started after the injury.”—*Caregiver #2, Wife of a man living with SMI*
Drowning	Descriptions of or concerns about the person with SMI drowning or nearly drowning	“I: When did you notice something is wrong with him? R: It was seven years ago. Something pulled him into the water. I think it is Satan. Myself and the whole family believes that Satan lives in the river. What else could it be that can pull him in to the water?”—Caregiver #12, *Father of a man living with SMI (secondarily a person living with SMI* “R: I love her. She is my daughter. I do not hate her for her illness. She is my responsibility. Her worry is my worry. Her illness is my responsibility. I sometimes feel sick because of the stress of caring. I lose [my] balance to stand still sometimes. I hide when I go to attend social gatherings like mahaber [association with or without religious intention for the purpose of social support and recreation] and idir [burial association]. She does not control me much in this regard. However, whenever I leave the house, I have a fear that she might hang herself or drown into water. She has never tried anything like that to date. She never tried to harm herself.”—Caregiver #13, *Mother of a woman living with SMI*

*I* interviewer, *R* respondent

**Table 2:** Other stressful or scary events that caused emotional suffering or distress

Code	Description	Examples
Restraint	Descriptions of or concerns about the person with SMI being tied up, chained, restrained, or kept inside or hidden involuntarily	“I: There are caregivers at the holy water place. R: Yes. He was tied with shackle… and his feet got worm. Finally his feet got worm… he got wound. Yes. He got wound and worm.”—*Caregiver #9, Mother of a man living with SMI* “I: Have you ever tried to chain her or shackle her? R: No. Never. But I kept her in a locked room. I still lock her in sometimes. Sometimes, she drives me really crazy until my mind works no more. Then I keep her inside and keep the door locked. Don’t you ever think she stays quiet when locked in. She knocks from the inside nonstop. Then I open it and give her coffee and food. She eats. This is how my life is.”—*Caregiver #5, Daughter of a woman living with SMI* “R: They took me to the holy water when they noticed that I was ill. I: were you chained? I: Yes. I was chained for some three days. They had to put me in chain when I bothered them. I don’t remember details but they told me that I was chained. I was first chained, then taken to holy water and finally went to the hospital.”—*Person Living with SMI #6, Male* “I: Have you or your son been chained up? R: I had been chained up for long. My wife used to tie my hands with a rope. My son has never been chained up because he never left the house. He isolates himself when the illness comes.”—*Caregiver #12, Father of a man living with SMI (secondarily a person living with SMI)*
Verbal or emotional abuse	Descriptions of or concerns about the person with SMI experiencing verbal or emotional abuse	“R: No, rather it gets me angry. If I had to go get something from the kitchen, she may destroy something for the time she wasn’t supervised. My job is to attend her. When I get angry, I tell her I will kill you and then kill myself. That is when I feel so bad. I really feel bad about myself but I never imagined other people looking me down.”—*Caregiver #5, Daughter of a woman living with SMI* “R: No one would take him seriously. They do not consider him equal with themselves. Even in the family, they do not consider him seriously. Everyone ignores him or whatever he would say. It doesn’t matter if he is talking about something good or bad, no one listens to him.”—*Caregiver #11, Father of a man living with SMI* “R: Some people call me crazy. I just forgive her because I know that I am not crazy myself. Some woman in my neighborhood calls me a goat. I do not try to argue with her because arguments are not good for my health. My mother is my confidant. I talk to her and she would tell me to ignore such people.”—*Person living SMI #4, Female* “I: Have you ever been chained? Abused? R: I have never been chained up but people say to me all sorts of things. They like to annoy me and that worsens my illness. The insults and curse words they say to me are not many after I received the treatment. But there are still some people who like to tease me. I just try to ignore them. What more can I do? What power do I have? When I tell them about my illness, they underestimate it and tell me that I will be ok.”—*Person Living with SMI #1, Male*
Exploitation or Disenfranchisement	Descriptions of or concerns about being forced or unpaid labor or being taken advantage of	“R: I think a lot about his mental health and what other people had done to him. They fool him to work for them for free. I could have sued them but I want to live in peace with my neighbors. I enter into fights with people when they let him work for them for no pay. I know there is a law to protect him but I did not want to fight with all the people who I respect. Over the past two years, he has not worked for me. He did not even change his clothes. He has no friends.”—*Caregiver #7, Father of man living with SMI (previously husband of a woman living with SMI, who has since died)* R: “I have no one to think of from our neighbors. If there should be someone held responsible, it is my sister in law. All my mother’s land ownership documents had been transferred to my brother. We had a fight with my brother because he cannot be the only one to inherit. We also asked him to support our mom as he owns the land.”—*Caregiver #5, Daughter of a woman living with SMI*
Forced treatment or care	Descriptions of or concerns about being forced into or forced to take treatment against the person with SMI’s will	“R: He won’t agree with me. He won’t go. Last time I took him, we rented a Bajaj [motorized rickshaw]. The Bajaj waited for us by the main road. The driver helped us to put him in. My daughter was with me. There was also another man, a relative of ours. We took him but he was not willing. We had to struggle every step of the way. He would not cooperate with anyone but that day he was submissive. But he got nervous when people held him to put in the Bajaj. He was soaked in sweat. Then we tried to convince him that we are actually taking him to the market to buy him clothes. He believed our relative when he said he will buy him clothes and went in the Bajaj with him. Then the Bajaj dropped us off at the health center. He refused to sit there. May God bless the health professionals. They did not keep us waiting. They asked me different questions. Then they gave me some money to buy soap.”—*Caregiver #4, Mother of a man living with SMI* “R: Yes I went with him. They gave him tablets. I tried to give him but he refused. He did not cooperate to swallow the pills. I tried to dissolve it in the water he drinks and also hide it in his food but he did not want to take it at all. I think the pills could make him feel better but he resisted. The pills really helped but he resisted. What can I do if he refuses to take? He doesn’t want to take anything at all. He gets irritable and tries to beat me when I tell him to take his pills. I tried really hard to help him finish the first round of tablets he was given by dissolving his daily dose in his tea, coffee or water. But I did not go back to get him the refills because I can’t continue doing that as he kept on refusing. He took his medications for a month.”—Caregiver #1, *Mother of a male living with SMI* “R: His brothers live a bit far. I do not have anyone else close by who can help. Taking him to the health center could have been easier if he was chained up and shackled. He might disturb the cart driver otherwise. The cart boys do not want to take someone with mental illness. That was why they raised the money to 80 birr last time.”—Caregiver #14, *Wife of a man living with SMI*
Chased or run off	Descriptions of or concerns about the person with SMI being chased away	“I: Generally speaking, what should be done? R: There should be counseling. Someone should work with him and his mother so they can be at peace again. I tried to counsel him but he does not listen. I even chased him out of the house when he does not listen but he slept on the streets. I do not know what to do. The injection also makes him look disfigured. He would sleep till 3 pm when he was taking the tablets. He does not eat well even after he wakes up. He won’t be able to eat if he does not work because we do not give him food. We do not have enough food for ourselves. He is 19 years old. He should be able to support himself.”—Caregiver #12, *Father of a man living with SMI (secondarily a person living with SMI*

*I* interviewer, *R* respondent

### Interactions between trauma exposure and mental illness

**Fig. 1:**
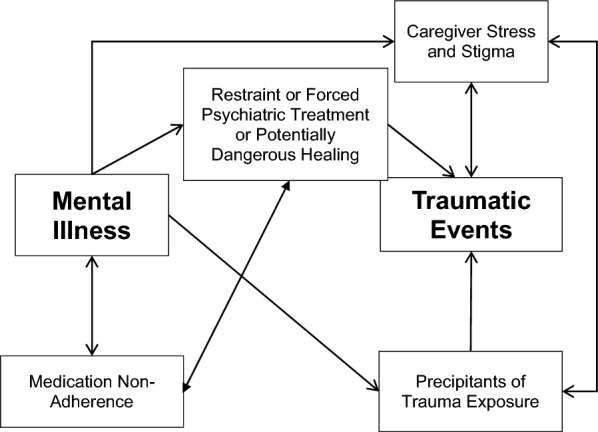
Model of identified interactions between traumatic events and mental illness

Participants described several ways in which they thought that trauma exposure caused or exacerbated mental illness, and ways that mental illness led to more exposure to traumatic events. These pathways are illustrated in Fig. 1.

### Traumatic events as a cause of mental illness

Some participants reported that trauma exposure was the cause of the mental illness that persons with SMI were living with. For example:

“When he was going to Dire Dawa with his wife they had a car accident where his wife survived and he remained like this. He just had a minor injury on his arm. After that accident, he has never been normal.”—*Mother of a man living with SMI* #1

“He complains that he has mental illness. He says something worries him a lot. It has been around seven years since this has happened. The cause is that he had an accident when he was riding a motorbike to one of the rural villages. He broke his leg and the accident was a major one. The illness started after the injury.”—*Wife of a man living with SMI* #2

### Traumatic events exacerbating mental illness

Persons with SMI also reported that their mental illness was worsened by traumatic experiences:

“I have never been chained up but people say to me all sorts of things. They like to annoy me and that worsens my illness.”—Man living with SMI #1

“The neighborhood is a tough place to live in. Sometimes they may call and curse you for no reason. I pretend as though I don’t see or hear what they had to say. Their words wound our hearts…. Sometimes, when people say bad things to me, I feel like harming myself but the thing is there are small children I care for.”—Woman living with SMI #2

“I was tied with some garment strips. My mother used to tie me with her scarf. That was just for a day. My brother beats me up occasionally. Even now, my nieces and nephews beat me every day. Some other children from the village also beat me. They call me a ‘slave’. It makes me so sad.”—Woman living with SMI #3

### Traumatic events experienced by people with SMI increasing caregiver stress and distress

In addition, caregivers reported that their own mental health was compromised by worry about people with SMI experiencing traumatic events.

“I complain a lot about life. That is affecting me psychologically. Every time I hear something bad about him, my mind gets disturbed. The anger from hearing what other people do to him is affecting me every day. At times my mind does not think straight. I cannot work due to anger but there is no one to understand that and help me… My livelihood is jeopardized after his illness.”—*Father of a man living with SMI* #3

“The tablets are good to keep them at home. I am happy with the very fact that they can sleep. They don’t go anywhere else. They don’t wander around. I don’t fear that the hyena might have eaten them. I can sleep since they too sleep. I wouldn’t have been able to rest if it wasn’t for the medication. They used to disturb me a lot. I could barely sleep before. Now we sleep in peace.”—*Mother of a man living with SMI* #4

### Precipitants of trauma exposure: mental illness increasing vulnerability to experiencing traumatic events

Participants, both caregivers and people with SMI, described many ways in which they thought that mental illness increased people’s risk of experiencing traumatic events. Specifically, they noted situations in which mental illness (a) led people to be more aggressive or violent, (b) resulted in people wandering off, getting lost, or becoming homeless, (c) led people to misuse drugs or alcohol, or (d) or led people to disrobe in public (see Table 3). Moreover, these vulnerabilities were perceived to be further exacerbated by (e) medication non-adherence or disengagement from mental health care.

**Table 3:** Factors perceived to increase the likelihood of exposure to traumatic events

Factors that might lead to trauma	Descriptions or concerns about factors that may be associated with risk of experiencing trauma	
Homelessness	Descriptions of or concerns about living without shelter	“R: I will take care of her until I die if I can. If I can’t, I would just let her go to the streets. I: Where would she go when you leave her? R: She would go on the street talking to random people. She would greet people she does not even know and talk to them haphazardly. She may suggest to go with them. She is a typical crazy woman except we kept her at home this far.”—*Caregiver #5, Daughter of a woman living with SMI* “I: Have you ever been homeless? R: I had an experience of homelessness when I was young. That was so many years ago. I sometimes feel like that was what caused me the mental illness.”—*Person Living with SMI #1, Male*
Wandering off	Descriptions of or concerns about leaving home without notice to family or friends	“I: Is the medical treatment good for ****? Is the medicine good? R: Yes. It had helped him recover. I: What changes did you see after he started taking the medicine? What are some of the changes? R: He had recovered very much. But like I told you, I stopped it. I: OK. What are some of the changes you saw? R: Before, he disappeared in the bus station a lot of times. He went up to Kella He went to Kella on foot and someone brought him to me. He has also went to Awash many times, he had disappeared and I have seen many troubles… because his mind is not right. I: Now since he started the medicine R: He is fine. He sits at home. He does not go anywhere else.”—*Caregiver #9, Mother of a man living with SMI* “R: I brought her here recently and she had an injection. I paid 150 birr [$6.70 USD] then we had a relief for 5 days. She was so calm. I don’t know, maybe the medication made her feel tired. We were so happy and thanked God. But now she started the urge to go again. She just wants to go somewhere. When she wants to go wandering around, she does not know even that she has to come home in the evenings.”—Caregiver #5, *Daughter of a woman living with SMI*
Aggression by a person with SMI	Descriptions of or concerns about a person with SMI being violent or aggressive to others	“R: In addition to money, I also need someone who can help me handle him when he is ill. Handling him all by myself is not easy. He cooperates when he is better but sometimes managing him gets really tough. He does not sit at home stable. That was how my mother left him. He sometimes tries to beat people. He may try to slaughter someone. He does not try to attack me but he was very violent towards my brother.”—Caregiver #15, *Child of a man living with SMI* “I: Does he try to harm others or break things? R: Previously he used to break things and beat people. He is not aggressive anymore. I: So why did you have to lock him in? R: We do not keep him in a padlock. We just close the door from outside. He could have gone outside if he wants it but he sleeps mostly. There are no people coming to visit him. He used to insult people when we leave the window open but he is quiet these days. He is tired mostly. He may have other health problems we do not know. We just think of his mental illness but I suspect that he may have another illness.” Caregiver #6, *Sister of a man living with SMI* “I: How do you see the treatment in general? R: It is good. If it wasn’t for the tablets, I wouldn’t have been able to function. I would have entered into fights with people when they say nasty things to me. The medication adds patience to people.”—Person living SMI #4, *Female* “I: Is he violent? R: He used to be very violent. Now he is calm. The only time he gets violent now is if we try to have him treated, wash his body or change his clothes. His body has layers of stain but we can’t do anything about it. We are just here to sit and observe.”—Caregiver #14, *Wife of a man living with SMI*
Psychiatric Illness	Descriptions of or concerns about psychiatric symptoms	“R: It is only mental illness. The illness made him talkative. He leaves the house during the dark and wanders around. He talks aimlessly. He is aggressive at times and doesn’t listen to us at all. He walks around talking nonsense. He doesn’t listen to us. I: How long has it been? R: It is about four years now. R2: It has been long. He talks through the night and keeps on talking during the day. The illness makes him wander around a lot for no reason. He talks to everyone on the street.”—Caregiver #1, *Mother of a male living with SMI* “R: It is a difficult situation to explain. It first started when I was drinking from river water directly just like the animals. After the illness, some weird thoughts crept into my mind. I started to think like I am related to Mengistu Hailemariam or Meles Zenawi. I started to feel like I am Jesus Christ. I felt like I am God. I became quite irritable and started saying rude words to people. I try to make peace with them when my illness goes away. I insult people when I am ill. I don’t remember some of the issues I said to people. the treatment has helped me a lot. I tried the holy water but it did not heal me. It might have worked for other people but that wasn’t the case for me.”—Caregiver #12, *Father of a man living with SMI (secondarily a person living with SMI)* “R: When it first started, he said there is something burning in his head. He said his mind is burning and started crying non-stop. Then we took him to Meskelo district where there is treatment. We put him in chain and took him there via Zway because it wasn’t accessible from our side.”—Caregiver #14, *Wife of a man living with SMI*
Alcohol or drug misuse	Descriptions of or concerns about alcohol misuse	“R: He will stop taking the medicine saying he is fine and wants to go to social settings with other people. Then he goes out and drinks with other people. I: Would being on medication prevent him from being in social settings? R: What do I know? I: Or are majorities of his friends those who like alcohol? R: His friends won’t even pick him up say he fell. They don’t want him to be well and healthy. They want him to be laughed at or to die. They want him to be left on the street. I have kept quiet not to fight with all his friends. I just receive him whether he is drunk and brought to home by other people or he walks himself. He was not well even last night. I was trying to remind you about the illness when I noticed some symptoms but he is fooling me. I just have to be tolerant. What more can I do? I: So in your opinion Mr ****’s problem is mainly alcohol? R: Yes.”—Caregiver #16, *Wife of a man living with SMI* “R: Yes. We do not drink coffee with our neighbors. Nobody comes to our house because they are scared of him. For example, the woman he had beaten the other day has never returned to our house. We do not go our neighbors’ house either because he might follow us if we go to them. For some unavoidable social gatherings, we have to wait until he goes somewhere so we can go to them for a brief stay or one of us has to stay behind with him. He would go to people’s houses and sit outside demanding for food and drinks. He would go and ask for arakie [a local spirit drink] if he smells people making it. He would use force to get what he wants. We feel ashamed of his deeds but he does not feel that way.”—Caregiver #8, *Father of a man living with SMI* R: “My children do not want to come close to him because he beats them. Other people also do not want to help me because he might get violent towards them. Currently, there is someone who is holding his both hands behind so we can cut his clothes and dress him up with another clean cloth. From all our neighbors, there is only one person who is helping me with that. We do not have anyone else as people are scared of him. Even when he beats me and when I cry out for help, nobody would come to rescue me. My children would run away to protect themselves. He would hit people with anything he could find. He will throw things on us. He would go even crazy when he chews khat. He wants to slaughter people when he chews khat. People give him khat knowing that he is mentally ill. He would be violent towards us every day he chews khat.”—Caregiver #14, *Wife of a man living with SMI* “I: Does he drink? R: He does. He chases me out of the house when he drinks alcohol. He insults me really bad. He drinks mostly around paydays. Around that time, he chases people on the street and throws stones at people. His colleagues tell me to advise him not to drink but he does not listen to me. I meet his colleagues when I go to receive his salary. The office decided to pay me (the custody 100 birr) instead because he is not well and not giving me. They threaten me that they will stop his salary because he is bothering them at work. My mother used to receive his payment before but now I go to receive his payment. They do not want to talk to him. They pass that responsibility to me instead. That is worrisome for me. I try to sit him down and talk to him but he said he would even stop the job. I tried to tell him that he needs the money. He would listen to me and apologize when he is feeling better.”—*Caregiver #15, Child of a man living with SMI*
Public nudity	Descriptions of or concerns about being naked in public	“I: Who was helping you around the time you heard that your father walked naked? R: There are two men in town. They came to school and called me from class to tell me that my father tore away his clothes and is walking naked. I was so disturbed when I saw him. We brought him home and they clothed him.”—Caregiver #15, *Child of a man living with SMI*
Medication non-adherence	Descriptions of or concerns about medication non-adherence	“It is a known fact. The illness is going to come back. I will bring him medications then. You know we see the difference in just two days. I am willing to get him his refills tomorrow but he won’t take it. He is not a child. I can’t beat him to take his medication. I can only advise him.”—Caregiver #16, *Wife of a man living with SMI* “It has been four months since he stopped taking the medication. He stopped taking the tablets because he said the tablets are making him weak. He started fighting with his mother. He has never been anywhere else after he started the treatment.”—Caregiver #12, *Father of a man living with SMI (secondarily a person living with SMI)* “R: Once he provided all information correct. The doctor asked him different questions and he was correct in answering. Then the doctor prescribed him medications. He returned to his uncle’s house with tablets. He was chained there. He felt better with the tablets. I stayed with him for five years. Two of my children were born after he got mentally ill. Later, he refused to see me. He chased me out. I went to my mother’s house with my children and stayed there for nine years. Caring for him was tiresome for me. All his brothers are also tired. We took him to different holy water places. There is nothing left that we could do. It has been seven years since we stopped everything we do to help him. He has not been on medication ever since. He refused all medications. Recently we heard that there is treatment at the health center. We rented a cart to get him there. We had to pay 80 birr. My two sons were trying to help him board. He beat them both and escaped. My son passed out and that shifted our focus.”—Caregiver #14, *Wife of a man living with SMI*

*I* interviewer, *R* respondent

### Traumatic events occurring in the context of seeking mental health treatment or healing

In addition to mental illness increasing vulnerability to experiencing traumatic events, some participants described persons with SMI experiencing traumatic events during religious or traditional healing or when being transported to receive psychiatric treatment at a clinic or hospital. The following participant describes being beaten by religious healers and fleeing into the jungle to escape the beatings, where she was at risk of harm from animals. When she returned from the jungle she experienced further beatings by her family members:

“I was also taken to St. Gabriel holy water. There was some religious ceremony taking place around my family’s house. I decided to return home and attend the ceremony instead since some guy was beating me so hard. The priests were also beating me with the cross asking “who are you?” as though I were a spirit. It was painful when they beat my forehead with the cross. Because they expected me to shout, I shouted saying my name out loud. I said, “I will leave, fine”. But there was no spirit left me when we went to St. Urael, another holy water place. I was baptized at Urael holy water but nothing changed. Then I left the place and stayed four nights in the jungle with the hyena. I think the hyena was full, he did not want to eat me. I stayed at the holy water for a week. My mother also stayed with me but she decided to return home without me. She was shocked when I followed her home. I was being beaten at the holy water…I have partial paralysis from their beatings.—Woman living with SMI #3

In addition, due to untreated active psychosis and other SMI symptoms, informants described situations in which some people with SMI were restrained in order to be forcibly taken to health clinics or other treatment facilities. This forcible restraint was sometimes reported to be terrifying for, or harmful to, the person with SMI.

“We took him but he was not willing. We had to struggle every step of the way. He would not cooperate with anyone but that day he was submissive. But he got nervous when people held him to put him in the bajaj [motorized rickshaw]. He was soaked in sweat. Then we tried to convince him that we are actually taking him to the market to buy him clothes.”—*Mother of a man living with SMI* #4

“A patient had come… for instance tied… their hands tied behind their backs, and bleeding—they had come…. Because they are tied very tightly … they were bleeding. They come as such, mistreated.”—*Male health care provider* #1

### Traumatic events due to caregiver desperation, stress or stigma

Lack of availability of treatment was reported to have led to many people with SMI being chained, shackled or restrained because of caregiver concerns about the safety of the person living with SMI or the safety of the community.

“My mom wants to beat people for every single thing…. We are not sure of what to do about her. When we run out of choices, people told us to restrain her at home because she may also be hit by a car or fall into a well. Now we locked her in the house. I kept her in a locked room. I still lock her in sometimes. Sometimes, she drives me really crazy until my mind works no more… Sometimes, when we have guests over, our guests question what is going on because she makes noises and knocks on the door from the inside. There have been times when people passing by tried to figure out what is going on. When they try to do that they disturb us. That concerns my husband.”—*Daughter of a woman living with SMI* #5

“When the illness starts he does not communicate to people. He fights with people sometimes. He doesn’t like me. He lives behind closed doors without a toilet. He pees in that room. We give him food through the window. We put the food in a plastic bag and throw it in for him… The door to his room will remain always closed unless my husband wants to go in and clean his room…It has been a year since we put him in a closed house. He used to be fine when he was getting the treatment from Amanuel.”—*Sister of a man living with SMI #6*

In some cases, caregivers reported beating or restraining people living with SMI because of stigma and fear that the person’s mental illness would be discovered by other people in the community.

“I used to be a known rich man in this kebele [administrative unit similar to a neighborhood]. First my wife got sick. I tried to hide her illness. I used to beat her to keep her at home so no one knows. I succeeded to keep her illness as a secret for 11 years. Later [my son] started acting strange. I was beating him to behave well.”—*Father of man living with SMI (previously husband of a woman living with SMI, who has since died)* #7

Participants, both caregivers and people living with SMI, reported that caregiver stress decreased when persons living with SMI were adherent to their medication and their vulnerability to traumatic events decreased. Engagement in treatment was often the primary reason a person was no longer chained or hidden away.

“They put me in chains and kept me at home. That was some 15 years ago. Then I started shouting at home. I shout at people to give me food. When they got tired of me, they put me in chains. Then I went to Amanuel hospital. My neighbors took me to Amanuel hospital. There I found my medication. I have not been ill again in 15 years time.”—Woman living SMI #4

### Complex interacting relationships between traumatic events and mental illness

As illustrated in Fig. 1, participants described many complex interacting relationships between traumatic events and mental illness. These included exposure to traumatic events leading to the onset or exacerbation of mental illness, and mental illness leading to experiencing traumatic events by increasing the likelihood of precipitants of exposure, dangerous or forced treatment, or caregiver stress and stigma. In addition, participants described experiences in which they perceived that dangerous or forced treatment resulted in more medication non-adherence, and in turn, medication non-adherence also seemed to lead to situations that put participants at risk of trauma exposure. Finally, caregiver stress and stigma not only directly resulted in increased traumatic events through increased use of chaining/restraint and beatings or assaults, but also increased the likelihood that people with SMI would experience precipitants of trauma exposure such as substance use and homelessness [51].

Often the interactions between these factors for each individual person with SMI were complex. For example, the father below describes the interactions between the mental illness symptoms of his son and trauma that his son is experiencing including being chained, being forced to receive treatment, and as a result, fleeing and being seriously injured. The situation described was exacerbated by the son’s medication non-adherence, alcohol misuse, and stress and hopelessness of the caregivers, which had resulted in two suicide attempts by the mother and concern on the part of the caregiver that he may kill his son or his son may kill him:

“The severity of his illness increased over the past six years. In 2012, he got seriously ill around midnight once. He escaped from the house and went to someone’s house. He was seriously injured when we found him. Then I took him to Amanuel.… He was fine when he was taking the medication from Amanuel but now he slipped into his alcohol again and he refused to take the medication. He forces people to give him drinks and sometimes he begs around for alcohol when there are some celebrations in people’s houses…. He drinks more than a bottle a day. His mother attempted to hang herself twice because she was hopeless about his situation…. I also tried to apply to the police station to keep him at prison because I could not handle him myself. They said it is not appropriate to request the assistance of the police. My son is so strong and aggressive. Apparently, I am old and I cannot handle him by force. I am accountable for any damage he does to other people and their properties. Chaining him is the responsibility of the family. The police said they need evidence to put him in prison.… What if he kills someone and I end up in jail? What if I kill him and go to jail instead? Where else can I go? Who would take care of my children if he kills me?”—*Father of a man living with SMI* #8

## Discussion

In this study, we investigated traumatic experiences for people with SMI living in a low-income country, and the relationship between trauma exposure and SMI symptoms. Multiple types of traumatic events were identified including those that met DSM-5 criteria for traumatic events such as beatings, assaults, being mugged or robbed, motor vehicle accidents, drowning, falls, and electric shocks. Notably although these experiences were reported to be common in Ethiopia and meet DSM-5 criteria [39], some of these events such as animal attacks, falls, and drowning do not appear on many commonly used trauma event checklists that were developed in the US such as the Life Events Checklist for the DSM-5 (LEC-5) [52], the Trauma History Questionnaire (THQ) [54], and the PTSD Checklist for the DSM-5 (PCL-C-5) [55]. In addition, several events were identified as being traumatic and were associated with subsequent mental or behavioral health problems, but did not meet DSM-5 criteria, such as being restrained or chained, being exploited or disenfranchised, verbally or emotionally abused, or chased away. These experiences have also been identified in a different sample of individuals with SMI living in Ethiopia [39]. Although many definitions of trauma used by the fields of psychology, psychiatry, and medicine would not classify these types of events as traumatic, the participants in this study noted that these were clearly traumatic events, in that they were extremely distressing and violating and resulted in long-lasting harm and a host of negative sequelae. Moreover, these upsetting events were associated with PTSD symptoms in another study of people with SMI in Ethiopia [39].

Indeed, research from India has found that despite exposure to events such as sexual abuse, individuals with SMI who were homeless or at high risk of homelessness identified traumatic events that related to social relationships such as alienation, shame, abandonment and rejection, and physical and verbal/emotional abuse from within their communities or own families as being the most distressing events they had experienced [56]. The authors suggest that cultural and contextual factors dictate which event are considered traumatic. For example, divorce in India, which occurs in less than 1% of cases, was considered traumatic by participants, in part due to the high stigma, broad social rejection, substantial decline in standard of living, and loss of children and close others that occurred due to divorce [57]. Many of these traumatic experiences were noted to have occurred because individuals had mental illness [56], which was also found in this study. The “mismatch” or “credibility gap” [58] between an individual’s self-reported experience of traumatic events and mental health professionals’ understanding of what constitutes a traumatic event, may be resulting in under-assessment and underreporting of the traumatic experiences of people with SMI [56] and subsequent underappreciation and undertreatment of trauma-related symptoms by health care providers.

Additionally, participants in this study reported that some attempts to address mental illness symptoms were traumatic in and of themselves, including being conveyed to treatment forcibly or to enduring dangerous or violent religious or cultural healing. Notably, participants did not mention forced medical treatment for SMI, including hospitalization and use of seclusion and/or restraints, as traumatic experiences, although they have been found to be traumatic in HIC settings [12–14, 23, 59–62]. The result of traumatic psychiatric treatment in HIC has been found to be associated with avoidance of care or disengagement from treatment [63, 64]. Potentially traumatic experiences of forced treatment or care may be particularly relevant in LMICs, which may have fewer protections against treating individuals against their will [65]. Unfortunately, there are very few data on emergency treatment of mental health conditions in LMICs [66]. Individuals in LMICs may also be more likely to frequent traditional or religious healers who may utilize potentially traumatic interventions such as shackling [67]. Healing or treatment that results in abusive or traumatic experiences, such as those described here, should be addressed, not only because they compromise the safety of the individuals, but also because they may exacerbate mental illness symptoms, lead to trauma symptoms, or result in less willingness to engage in health care, including psychiatric care [59]. Efforts that have been made to have mental health professionals collaborate with traditional healers may present opportunities to reduce the use of harmful practices and prevent traumatic experiences [68].

The results of the study found that participants perceived that in many cases, SMI symptoms were the result of trauma exposure. This is supported by a large body of research from HICs that has found that traumatic events predict the development of psychosis [29–34, 69–74]. Despite these findings from HICs, much research on community beliefs about the etiology of SMI in LMICs has focused on supernatural or spiritual beliefs [75], and far less attention has been paid to community perspectives that trauma exposure precipitates the onset of SMI. Moreover, less attention has been paid to the role of social adversities, such as poverty, loss, and abandonment on risk of trauma exposure and subsequent development of SMI symptoms [56, 76, 77]. Not only is this a missed opportunity to identity and address a factor that may be exacerbating mental illness symptoms, but it is also a missed opportunity to bridge the gap in understanding the etiological basis of mental disorder that may exist between patient and provider. Patients may be more likely than providers to believe that SMI symptoms stem from supernatural or spiritual causes, and conversely providers may be more likely to believe that SMI symptoms are derived from biological causes [78–81], but according to the results of this study, they may both agree that traumatic events, socio-economic adversities, and structural vulnerabilities may play a role in the onset of SMI symptoms, which might lead to shared understanding, trust, and rapport.

Participants in this study, including both caregivers and persons living with SMI, noted that distressing events were associated with more severe mental illness symptoms, medication non-adherence, and higher risk of future traumatic events. Moreover, participants described situations in which both trauma exposure and SMI symptoms seem to have exacerbated caregiver stress, desperation, and lack of hope. In turn, caregiver distress seemed to increase trauma exposure for people with SMI in the form of beatings, assaults, restraint and chaining, verbal and emotional abuse, and being chased from the home.

Traumatic events may be resulting in poorer health outcomes for people with SMI through a variety of pathways. Indeed, research from HICs has found that people with SMI, despite being engaged in mental health treatment, often are not assessed or treated for PTSD, even though it is estimated that 25%-50% of people with SMI in HICs have comorbid PTSD [23]. This comorbidity is associated with poorer health outcomes and functioning for people with SMI [23]. These poorer health outcomes may be even more pronounced in LMICs in which trauma symptoms overall [82], and specifically for people with SMI, may be more prevalent than in some HICs [28, 35, 37, 38, 40–42], and yet there is much less access to mental health services [36] and more people are living with untreated SMI [83].

Given these findings, psychological and anthropological research on representative samples of individuals with SMI is needed to better understand the role of trauma exposure in their lives and on their treatment and symptoms. It may be that contextually-appropriate community-based psychological and psychosocial treatment for trauma exposure needs to be part of the standard of care for treatment for people with SMI [84]. Service providers may need more training and awareness raising about the role of trauma exposure on SMI symptoms and treatment engagement throughout the world. In addition, psychoeducation for family and community members about trauma and its effects, as well as increased economic and social support and resources for people living with SMI and their caregivers, may be necessary to help prevent future trauma exposure and improve health outcomes.

The results of this study should be interpreted within the limitations of the study. This study used secondary data analysis from a qualitative study in a specific population of individuals with SMI, their caregivers, and health service providers in Sodo District, Ethiopia, and may not be generalizable to other populations. However, the circumstances under which participants were living in Ethiopia, including high rates of poverty and subsistence farming, relatively low rates of educational attainment, limited social safety net and services, common use of traditional and religious healing for SMI symptoms, and difficulty accessing mental health care are very common across multiple settings around the world [85, 86]. Indeed 80% of the world lives in LMICs [87] and most LMICs don’t have adequate access to mental health services, particularly for individuals with SMI [36]. It is therefore likely that these findings will be relevant to multiple populations and settings. Indeed, the results are similar to those found in a study on individuals with SMI in Tamil Nadu, India [56, 57]. However, much more research is needed to investigate these relationships in larger and representative samples in LMICs and HICs alike.

## Conclusion

The results of this study strongly suggest that it is incumbent upon mental health professionals and the broader health community to view trauma exposure as a public health problem that affects all, and may be particularly relevant for individuals who have SMI. More research with representative samples of individuals with SMI is needed to better understand the role of trauma exposure in their lives, and to develop culturally- and contextually-appropriate psychological treatment for trauma exposure and comprehensive community-based support and resources for people living with SMI and their caregivers to help prevent future trauma exposure and improve health outcomes.

## Data Availability

The datasets generated and analyzed during the current study are available from the corresponding author upon reasonable request.

## Abbreviations

SMI: Severe mental illness
PTSD: Post-traumatic stress disorder
HICs: High-income countries
LMICs: Low and middle-income countries
PRIME: Programme for Improving Mental healthcarE
mhGAP: Mental health Gap Action Programme
OPCRIT: Operational Criteria for Research
DSM-5: Diagnosis and Statistical Manual-Version 5
DfID: Department for International Development
NIH: National Institutes of Health
NIHR: National Institute of Health Research
PTEs: Potentially traumatic events
